# Colossal permittivity behavior and its origin in rutile (Mg_1/3_Ta_2/3_)_x_Ti_1-x_O_2_

**DOI:** 10.1038/s41598-017-08992-x

**Published:** 2017-08-30

**Authors:** Wen Dong, Dehong Chen, Wanbiao Hu, Terry J. Frankcombe, Hua Chen, Chao Zhou, Zhenxiao Fu, Xiaoyong Wei, Zhuo Xu, Zhifu Liu, Yongxiang Li, Yun Liu

**Affiliations:** 1 Research School of Chemistry, the Australian National University, Canberra, ACT 2601 Australia; 2 School of Physical, Environmental and Mathematical Sciences, The University of New South Wales, Canberra, ACT 2601 Australia; 3 Centre for Advanced Microscopy, The Australian National University, Canberra, ACT 2601 Australia; 4 Fenghua Advanced Technology Holding Co. Ltd., Zhaoqing, 526020 Guangdong China; 5 Electronic Materials Research Laboratory, Key Laboratory of the Ministry of Education & International Centre for Dielectric Research, Xi’an Jiaotong University, Xi’an, 710049 China; 6 CAS Key Lab of Inorganic Functional Materials and Devices, Shanghai Institute of Ceramics, Chinese Academy of Sciences, Shanghai, 200050 China

## Abstract

This work investigates the synthesis, chemical composition, defect structures and associated dielectric properties of (Mg^2+^, Ta^5+^) co-doped rutile TiO_2_ polycrystalline ceramics with nominal compositions of (Mg^2+^_1/3_Ta^5+^_2/3_)_*x*_Ti_1−*x*_O_2_. Colossal permittivity (>7000) with a low dielectric loss (e.g. 0.002 at 1 kHz) across a broad frequency/temperature range can be achieved at *x* = 0.5% after careful optimization of process conditions. Both experimental and theoretical evidence indicates such a colossal permittivity and low dielectric loss intrinsically originate from the intragrain polarization that links to the electron-pinned ${\bf{M}}{{\bf{g}}}_{{\bf{T}}{\bf{i}}}^{{\prime}{\prime} }+{{\bf{V}}}_{{\bf{O}}}^{\bullet \bullet }+{\bf{2}}{\bf{T}}{{\bf{a}}}_{{\bf{T}}{\bf{i}}}^{\bullet }+{\bf{2}}{\bf{T}}{{\bf{i}}}_{{\bf{T}}{\bf{i}}}^{\prime}$ defect clusters with a specific configuration, different from the defect cluster form previously reported in tri-/pent-valent ion co-doped rutile TiO_2_. This work extends the research on colossal permittivity and defect formation to bi-/penta-valent ion co-doped rutile TiO_2_ and elucidates a likely defect cluster model for this system. We therefore believe these results will benefit further development of colossal permittivity materials and advance the understanding of defect chemistry in solids.

## Introduction

The search for new materials with colossal permittivity (CP, >10^3^) to be used in capacitors has been recently driven by a new dielectric polarization mechanism: the electron-pinned defect-dipole (EPDD) observed in rutile TiO_2_ through ionic-co-doping^1^. Such EPDDs arise from the defect complexes that were formed by synergetic co-doping of “donor” and “acceptor” ions, one of which on its own is a difficult-to-dope ion, such as In^3+^ ion^1^. Materials of this kind having EPDDs generally show frequency- and temperature- independent CP with a low dielectric loss^1–4^ in comparison with previous CP candidates e.g. CaCu_3_Ti_4_O_12_ (CCTO) and La_2x_Sr_x_NiO_4_ (LSNO)^5, 6^. Such a new dielectric polarization mechanism inspires new CP candidate materials to be potentially used in capacitors and permit further scaling advances in electronic devices^7, 8^, though other issues are still required to be considered, such as the insulating resistance (conventional ferroelectric/relaxor dielectric capacitors generally have an insulating resistivity over 1 × 10^11^~1 × 10^12^ Ω·cm)^1, 3, 9–12^. Actually, the formation of EPDD in the host material is complicated since the formation of defect states is generally strongly related to the dopant ions, and to the preparation approach and conditions. The understanding of EPDD is still at an early stage, with research to date being mainly focused on trivalent acceptor and pentavalent donor co-doped rutile TiO_2_^3, 9, 10^, although some primary works, such as Zn + Nb co-doped rutile TiO_2_^13^, have been done in bi-/pent-valent doped rutile TiO_2_. Defect structures are related to the ionic sizes, charges and electronegativity of the dopants. For example, in the M + Nb co-doped rutile TiO_2_ (M = Al, Ga, In)^1, 12, 14^, the size of the acceptor ion controls the polarization behavior, so that the EPDD effect is dominant in the case M = In, but not for M = Ga, and not even structurally possible for M = Al. Size effects of doping ions are found in almost all dielectric materials, e.g. in both binary oxide and ABO_3_ type perovskite^10, 15–19^. As the CP is determined by the defect rather than the average structure, the preparation approach and process conditions of the synthesis of CP materials are therefore critical, affecting the formation of defect structures and thus influencing the dielectric behavior. This makes reported dielectric properties of In + Nb co-doped rutile TiO_2_ very diverse^18–23^. Intrinsically, an excellent CP property with a low dielectric loss can be achieved mainly or at least dominantly from EPDD^20, 21^ or from the localized electrons associated with the coexistence of Ti^3+^ and Nb^4+^ presented in the samples^22, 23^. In contrast, CP behaviors with significantly higher dielectric loss that comes from the extrinsic polarization contribution, e.g. the barrier layer capacitor (BLC) effect^24–26^, were also observed. The different dielectric behaviors are at least partially related to different preparation processes, including the temperature and atmosphere used for synthesis, which is intimately related to defect structures such as the oxygen vacancies and reduction of Ti^4+^ into Ti^3+^. It is noteworthy that rutile TiO_2_ itself under certain process conditions exhibits the reduction of Ti^4+^ into Ti^3+^, resulting in delocalized electrons which give BLC effects with a considerably increased dielectric loss^27^. Therefore, it is imperative to continue to systematically investigate different EPDDs formed in different co-doping ion combinations and host materials, advancing the understanding of defect chemistry in solids.

There are few CP report for bivalent acceptor ions in co-doped TiO_2_ except for Zn + Nb co-doped TiO_2_ done by Hao *et al*.^13^, by which the authors mainly focused on the dielectric properties of (Zn, Nb) co-doped TiO_2_ with different forms, including rutile ceramics, amorphous films and anatase films, and only proposed the possible defects for CP effect. After further more work done afterwards, we realized that the complexity of defects in those ionic co-doped TiO_2_ is critical and the defect formation are the factors to determine the individual dielectric behavior. We therefore initiate a first study herein on the co-doping of Mg^2+^ and Ta^5+^ into TiO_2_ to create CP materials and gain insight into the detailed relationships between the defects and the dielectric properties. Ta^5+^ was chosen as the donor because it has a similar ionic size to Nb^5+^ but possesses one more electron shell. Hybrid density functional theory calculations suggest that “the larger distortion around the Ti^3+^ site in the Ta-doped TiO_2_ gives stronger localization of the electron to the Ti^3+^ site.”^28^. The benefit of the Ta was further supported by the superior colossal permittivity behavior obtained in In + Ta co-doped rutile TiO_2_. Considering the bivalent acceptor of Mg in comparison with that of the trivalent ones, it is of significance to investigate the defect related dielectric properties and the configuration of the defect cluster in this new system to provide further guidance and understanding of defect related dielectric behavior in bi-/penta-valent ion doped rutile TiO_2_ system. Defect analysis combined with density functional theory calculations was conducted to understand the dielectric mechanism in this bi-/penta-valent ion co-doped rutile TiO_2_ system in addition to systematic dielectric property characterization.

## Results and Discussion

**Figure 1:**
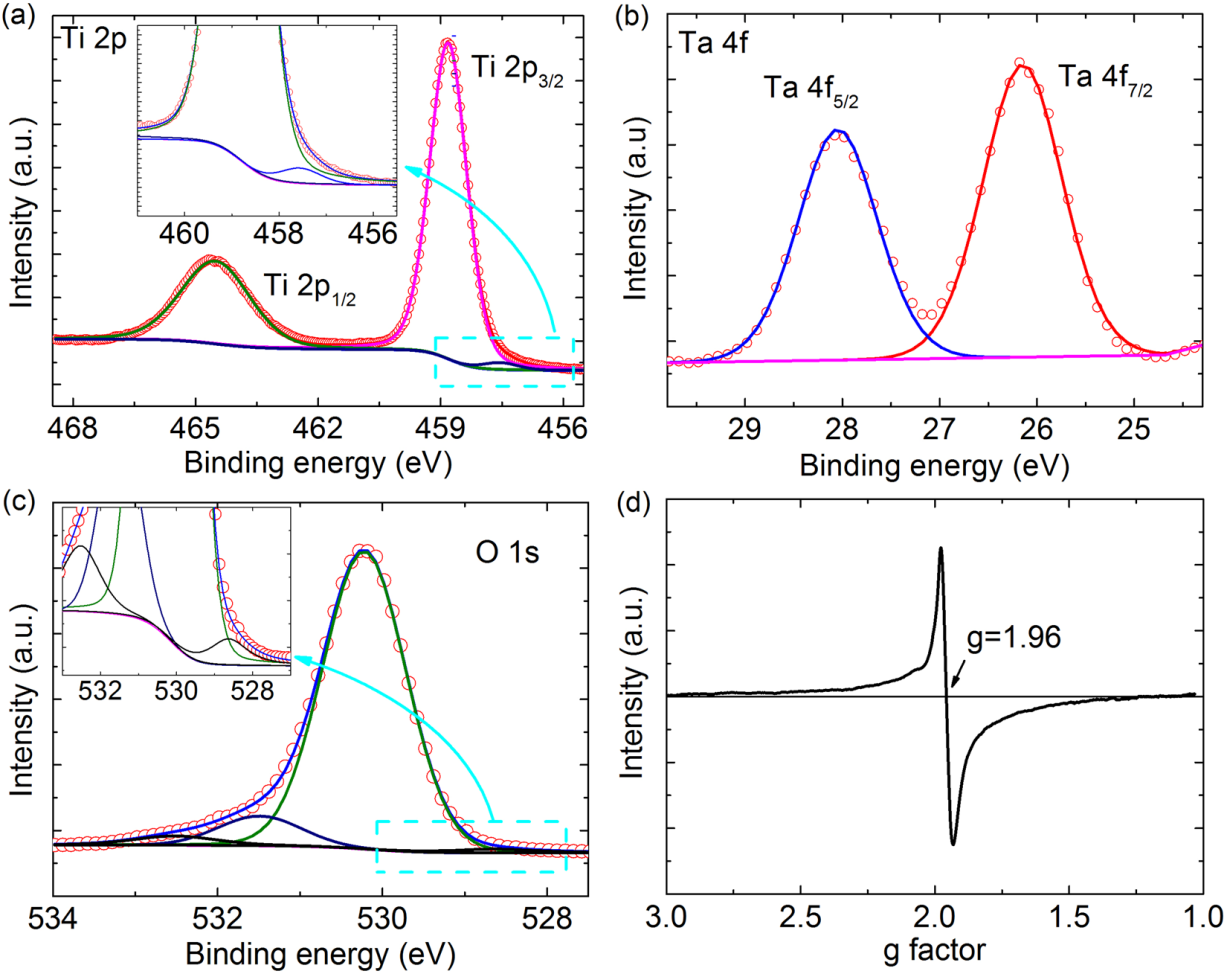
Valence states and defect characterization of Mg + Ta co-doped rutile TiO_2_. (**a**–**c**) Core level XPS (open circle) and corresponding fitting results (solid lines) of Ti 2p (**a**), Ta 4 f (**b**), O 1 s (**c**) electrons of 5% Mg + Ta co-doped rutile TiO_2_. (**d**) EPR spectrum of 0.5% Mg + Ta co-doped rutile TiO_2_ measured at 10 K. Insets in (**a**) and (**c**) are enlarged views to clearly show the shoulders in the blue dotted boxes.

The XRD patterns of the synthesized samples indicate that the average structures of all the samples are of pure rutile phase (Figure S1a). The average structures and micro-compositions were carefully examined by XRD and BSE with EDS analysis (Figure S1b–c). Further, the valence states of the Mg + Ta co-doped rutile TiO_2_, essential for uncovering the local structures/coordination environments as well as insight into the dielectric mechanism, have been analyzed using EPR and XPS techniques. XPS data measured on 5% Mg + Ta co-doped rutile TiO_2_ are shown in Fig. 1. The Ti 2p doublet (Fig. 1a) with 2p_3/2_ and 2p_1/2_ binding energies of 458.7 eV and 464.6 eV, respectively, is clearly present, corresponding to that of the pure rutile TiO_2_^29^. Slight reductions happen in the host TiO_2_, with a signal from Ti^3+^ evident in the low-energy shoulder, in addition to the dominant Ti^4+^ signals in Ti 2p spectrum. The presence of Ti^3+^ species in Mg + Ta co-doped rutile TiO_2_ was confirmed by the EPR powder spectrum. (Note that EPR can detect the unpaired electrons in very low concentrations-substantially lower than those detectable through XPS.) One signal with g = 1.96 was clearly detected in 0.5% Mg + Ta co-doped rutile TiO_2_, which is close to that of the values previously reported for Ti^3+^ ^30^. Figure 1b shows the presence of doublet Ta^5+^ 4 f peaks with binding energies at 28.0 eV and 26.1 eV, respectively. Their spin-orbit splitting of 1.9 eV is consistent with that of the Ta_2_O_5_ as reported in the literature^31, 32^. The Ta 4 f signal suggests that Ta has a normal state of +5 without any reduction. The O 1 s profile as shown in Fig. 1c has four components: a main peak at ~530.2 eV and a lower-energy tail at ~528.6 eV, corresponding to the bulk Ti-O and Mg/Ta-O cation-oxygen bond. Two further higher-energy peaks at 532.5 eV and at 531.5 eV are assigned to the presence of oxygen vacancies and surface hydroxyl, respectively^33^. These results suggests the Ta^5+^ to be responsible for the reduction of Ti^4+^ to Ti^3+^, which is consistent with previously reported results^9^ and also quite similar to that of the M + Nb co-doped rutile TiO_2_ (M = In, Ga, Zn), where Nb^5+^ substitutions locally induce the reduction of Ti^4+^ into Ti^3+^ ^1, 13, 14^. Therefore, EPDDs may also similarly form to account for the excellent dielectric properties, detailed below.

**Figure 2:**
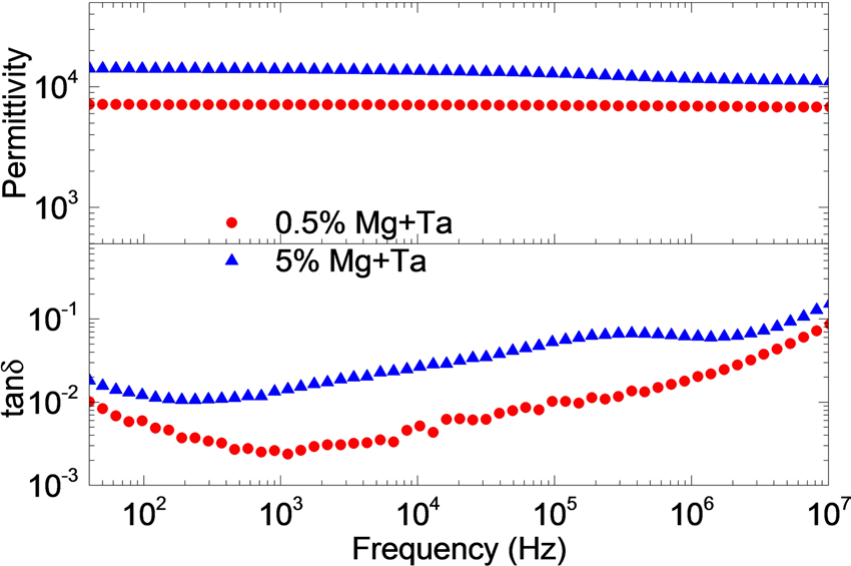
Dielectric permittivity and loss tangent (tan δ) for samples with 0.5% and 5% Mg + Ta co-doped rutile TiO_2_ with Ag painted electrode.

Figure 2 shows the dielectric spectra over the frequency range of 40 Hz to 10 MHz using an Ag electrode. As expected, colossal permittivity was obtained in Mg + Ta co-doped rutile TiO_2_. Increasing the doping levels induces higher permittivity. A small co-doping level of 0.5% created relatively stable colossal permittivity over 7000, with dielectric loss below 1% (e.g. as low as 0.002 at 1 kHz) in a broad frequency range up to 0.13 MHz, which is low compared with reported co-doped rutile TiO_2_ materials and also comparable to reported results for commercially used capacitors^1, 9, 13, 34, 35^. The surface effects were observed in 0.5% Mg + Ta co-doped rutile TiO_2_, with only small variations in dielectric properties using Ag or Pt electrodes (Figure S2a). For 5% Mg + Ta co-doped rutile TiO_2_, the permittivity increased by nearly a factor of two compared with that of the 0.5% Mg + Ta co-doped case, while also keeping a relatively low dielectric loss below (5% over a broad frequency range up to 100 kHz). However, there is a noticeable variation in permittivity corresponding to a relaxation peak in the 1 MHz region. This suggests that there was an external contribution from space-charge-induced surface BLC effect, supported by obvious variation in permittivity when using Ag or Pt electrodes (Figure S2b). The obvious surface effect suggests that the dielectric properties for samples with high co-doping levels become more complicated. The dielectric properties observed in 0.5% Mg + Ta co-doped rutile TiO_2_ are at least not predominated by the surface BLC effects based on less variation of dielectric properties under DC bias (Figure S3), as well as complex impedance analysis, which shows BLC effects only contribute to the dielectric properties at high temperatures, e.g. above 400 K (Figure S4a,b). These results are further supported by the frequency dependence of the imaginary impedance and modulus spectrum (Figure S5), where highly localized dipolar relaxation dominate in a broad temperature range. A long range hopping-conductivity-related interfacial barrier layer capacitor effect due to the excited electrons from defect clusters to grain boundaries and surfaces can be observed at higher temperatures (450 K), which together with the highly localized dipolar relaxation from intragrain contribution are responsible for the high permittivity behavior at higher temperatures, around 450 K.

**Figure 3:**
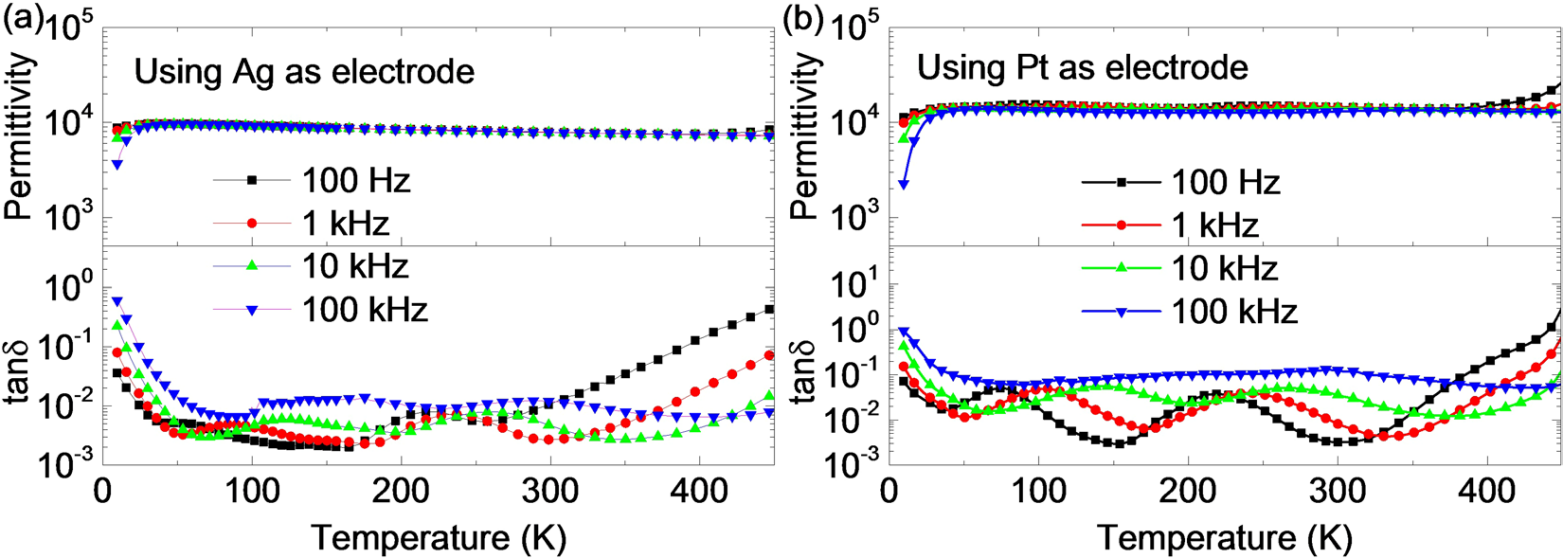
Temperature dependences of the dielectric permittivity and loss (tan δ) for 0.5% Mg + Ta co-doped rutile TiO_2_ with (**a**) Ag and (**b**) Pt electrodes.

Figure 3 shows the measured permittivity and dielectric loss from 10 K to 450 K for 0.5% Mg + Ta co-doped rutile TiO_2_. The CP (e.g. over 7000 for Ag coated samples in Fig. 3a) remains nearly unchanged from low temperatures (e.g. 20 K) to over 400 K. The overall dielectric loss is also quite small within a broad temperature range (50–400 K), where especially within 100 K to 400 K the dielectric loss can stay below 1% over the frequency range from 1 kHz to 10 kHz. The dielectric loss is as low as 0.002 in some regions. The main relaxation appears below 50 K, where the permittivity decreases while dielectric loss increases. The relaxation was not fully resolved, with the expected relaxation peaks being located at lower temperatures than the 10 K lower limit of this work. This relaxation was not dependent on the electrodes. Similar relaxations also exist in samples with higher doping levels. Thus, the main relaxation should be related to an electron freezing/activation process, similar to that of the relaxation occurring in the M + Nb (M = In, Ga) co-doped rutile TiO_2_, but here occurring at an even lower temperature^1, 14^. Though it is hard to figure out the activation energy, it reasonable to expect a lower value close to that reported in In + Nb co-doped rutile TiO_2_^1^.

**Figure 4:**
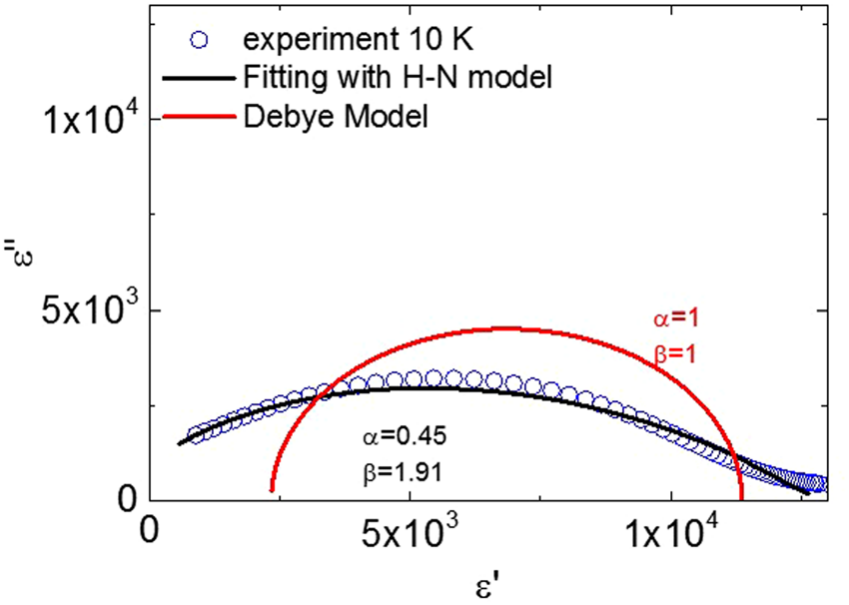
The complex permittivity (*ε* ^*^) plot of 0.5% Mg + Ta co-doped rutile TiO_2_ fitted with the Debye model and the Havriliak-Negami (H-N) model.

To further understand the nature of the dielectric relaxation, complex permittivity plot combined with analysis of charge hopping model was presented. Recently, Tsuji *et al*.^23^ suggest a hopping polarization to be responsible for the In + Nb co-doped rutile TiO_2_ samples synthesized by low-temperature spark plasma sintering (SPS) technique as the Havriliak-Negami (H-N) charge hopping model can reasonably explain their results. For conventional charge hopping model, their complex permittivity plot can be generally fitted using the H-N model^36, 37^. In the H-N model^38^, the *ε* ^*^(*ω*) can be expressed in a general form:

1 $${\varepsilon }^{\ast }(\omega )={\varepsilon }_{\infty }+\frac{{\varepsilon }_{s}-{\varepsilon }_{\infty }}{{(1+{(i\omega {\tau }_{HN})}^{\alpha })}^{\beta }}$$

where *ε*_*s*_ is the static permittivity and *ε*_*∞*_ is the permittivity at infinite high frequency, *α* the parameter for the peak breadth of the distribution of relaxation frequency and *β* the parameter for the symmetricity of the distributed function. Equation (1) reduces to an original Debye model when *α* = *β* = 1. The equation also reduced to Cole-Davison equation when *α* = *1* and 0 < *β* < 1. Figure 4 shows the fitting results with both Debye and H-N model. The Debye model fitting obviously leads to a bad fit. Using the HN model, the best fit was given with the parameters: *α* = 0.45, *β* = 1.91, time constant *τ*_*HN*_ = 2.32 × 10^−6^ s, *ε*_*s*_ = 626, *ε*_0_ = 12849. The fitting is not well simply from the plot and both the unreasonable value of the *β* and *ε*_*s*_ means that the conventional charge hopping model does not fit our case. Although both defect models, *i.e*. the EPDD proposed in this work and the hopping charge conduction in localized electrons in the distributed potential well proposed in refs 22 and 23, are quite similar, our case (no Nb^4+^) differs from the results reported previously^22, 23^ where the defects are associated with the existence of Nb^4+^ ions.

In addition to the main relaxation arising from the EPDD, two other relaxations (with much smaller amplitude affects) appear at ~50 K–150 K and ~160 K–330 K. Both are nearly independent of electrode, and also appear in samples with high co-doping level. The relaxation at ~50 K–150 K appears after the activation of frozen electrons. Again, similar relaxation has been observed in Ga + Nb co-doped rutile TiO_2_ and more strongly in Ta-only doped rutile TiO_2_ (Figure S6), and originates from Ti^3+^-related polaron-like structures. For these relaxations electrons are trapped by the distorted lattice structure giving local polarization relaxation behavior in the low temperature range, as experimentally observed in reduced rutile TiO_2_^27^, and theoretically demonstrated in Nb-only and Ta-only doped rutile TiO_2_^28^. The relaxation appearing at ~160 K-330 K exhibits obvious relaxation peaks in both the permittivity and dielectric loss spectra, a typical relaxor type relaxation behavior (as magnified in Figure S7), which is also observed in the same temperature range in *M* + Nb co-doped rutile TiO_2_ (*M* = In, Ga)^1, 14^ as well as In + Ta co-doped rutile TiO_2_^4^. This weak dielectric response with a permittivity of ~1000 could originate from ionic polar fluctuations. Both Nb-only and Ta-only doped rutile TiO_2_ have been reported to show an enhanced polarizability due to the easier distortion of the structure, both theoretically and experimentally^28, 38^. Moreover, slightly doped incipient ferroelectric materials are reported to show similar relaxor-type behavior arising from off-centre cation motion, where the polarizability of the phase increases as polar impurities are introduced^39–42^.

Given the excellent dielectric properties—especially the ultra-low dielectric loss (e.g. 0.002 at 1 kHz)—that were clearly present in this new material system, the structure of the defect dipoles was studied in more detail. To this end, density functional theory (DFT) computation was carried out on rutile TiO_2_, incorporating the detected and reasonably assumed ionic states, i.e. Ti^3+^, Ta^5+^, Mg^2+^, and oxygen vacancies.

**Figure 5:**
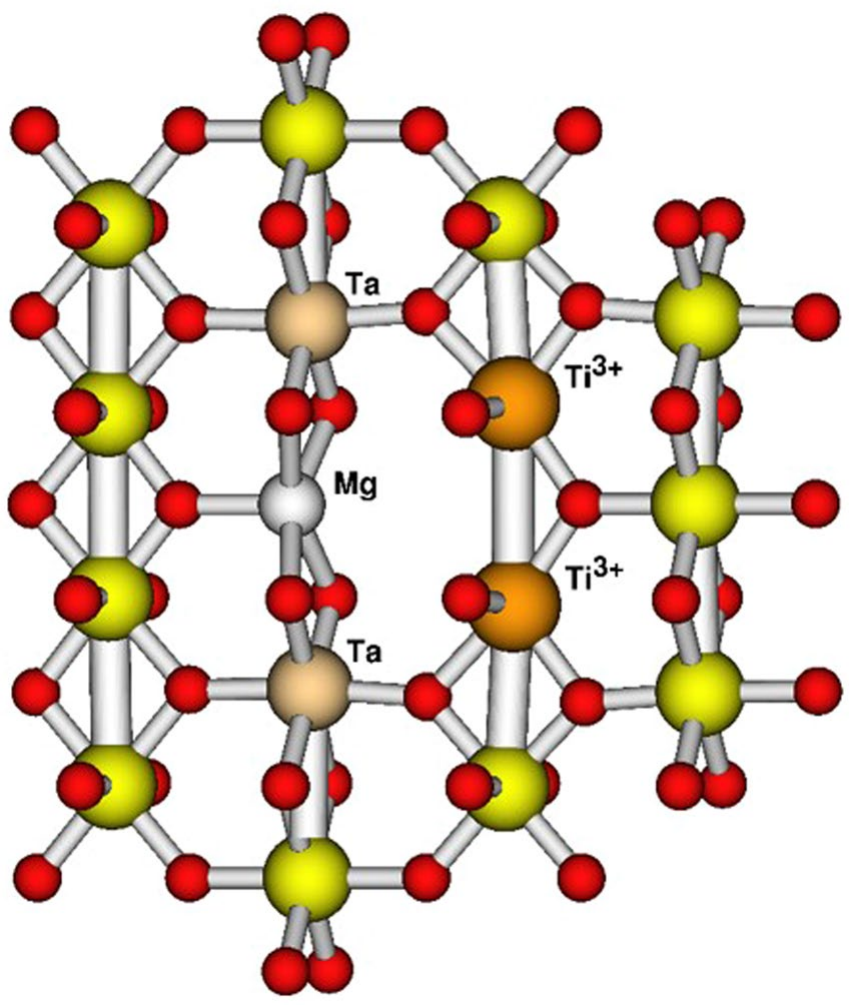
Ball and stick illustration of the lowest energy defect structure. Unlabeled atoms are Ti^4+^ (yellow) and O (red). Included octahedra lie in the 110 plane of the parent rutile cell.

Calculations were performed using the PAW formalism with the VASP code^43, 44^. Periodic boundary conditions were employed on 3 × 3 × 4 supercells of the rutile crystallographic unit cell. One Mg and two Ta ions were substituted for Ti in various configurations (corresponding to 4.2% co-doping). Straight substitution of Mg and Ta for three Ti ions produced substituted Mg^2+^, Ta^5+^ and no Ti^3+^, as might be expected. Only when oxygen vacancies were also incorporated were the experimentally-observed Ti^3+^ ions produced in the electronic ground state. There was a strong preference for oxygen vacancies to be located adjacent to the Mg ion; other locations yielded energies at least 1 eV higher than the most stable configuration. One Mg substitution, two Ta substitutions and one oxygen vacancy in the supercell usually resulted in two Ti ions reduced to Ti^3+^. Irrespective of the relative positions of the Ta^5+^ ions, at least one of these reduced Ti was adjacent to the oxygen vacancy. This is similar behavior to what has been observed in In + Nb co-doped TiO_2_ in our previous work, in which Ti is always reduced adjacent to the oxygen vacancy associated with two In substitutions^1^. The location of the second reduced Ti centre in the ground state DFT electron density varied depending on the relative positions of the Ta substitutions. Two defect configurations were found to be clearly lower in energy than any other investigated configuration. The lowest, illustrated in Fig. 5, minimizes the distances between the effective positive and negative charges (relative to the Ti^4+^ lattice) of the substituted and reduced ions. A second configuration with one of the Ta^5+^ ions shifted to occupy a Ti site adjacent to the other Ta and the Mg was found to give an energy around 0.2 eV higher. All other tested configurations with an oxygen vacancy adjacent to the Mg substitution gave energies around 0.5 eV to 0.7 eV higher than the most stable, giving the most stable configuration a significant thermodynamic advantage. However, it is important to note that even the non-thermodynamically-preferred defect structures maintained a reduced Ti site adjacent to the very stable Mg-V_O_ complex and another at most one octahedron away, leading to the EPDD effect and low dielectric loss. This result indicates that the EPDD with lowest configuration energy in this new material is quite different with that of the tri-/penta-valent ion doped rutile TiO_2_, suggesting that the CP behavior can be obtained from configuration-different EPDDs.

The DC resistance of the materials was measured with the sample thickens of 1 mm and diameter of ~1 cm. The resistance value was collected after the DC voltage was applied and hold for one minute. The resistance at low DC voltage (i.e. ~8 × 10^9^ Ω at 1 V) is quite close to that of the resistance calculated from the intersection of impedance semicircle on the real axis by the fitting (~9 × 10^9^ Ω). The range of this resistance is several order higher than the values reported in CCTO and LSNO (10^3^~10^6^ Ω) according to the intersection of impedance semicircle on the real axis for samples similar size^45, 46^. The DC resistance of the samples at high voltage (100 V) comes down to order of 10^6^ Ω, which is indeed far below the value of current disk capacitor and multi-layer ceramic capacitors^11, 47^. Further investigation is under the progress to figure out the dielectric polarization behaviors under a strong electrical field, which is beyond the scope of this paper. However, it is clear the CP with low dielectric loss below 1% in both Mg + Ta co-doped rutile TiO_2_ in combination with the reported low dielectric loss in In + Ta co-doped rutile TiO_2_ sample based on EPDD mechanism together provide the prerequisite for further development of these materials for use in capacitors, although there are still more works to be done to increase the insulating resistance of such CP materials.

## Conclusions

All the different co-doping levels in Mg + Ta co-doped rutile TiO_2_ tested in this work demonstrated CP behavior, while only the samples with relatively low co-doping levels could be well controlled to show excellent CP performance with low dielectric loss. Low frequency- and temperature-dependent CP (over 7000) with very low dielectric loss (below 1%, as low as 0.002 at 1 kHz) was observed over a broad frequency range from 40 Hz up to 130 kHz in 0.5% Mg + Ta co-doped rutile TiO_2_. The high performance CP behavior is dominantly from the intragrain electron-pinned defect clusters of $M{g}_{Ti}^{{\prime}{\prime}}+{V}_{O}^{\bullet \bullet }+2T{a}_{Ti}^{\bullet }+2T{i}_{Ti}^{{\prime}}$ with a specific form, rather than surface/grain boundary interface effect. A defect model was built for the first time for bi-/penta-valent ion co-doped rutile TiO_2_, which differs from those reported in tri-/penta-valent ion co-doped rutile TiO_2_ systems. This expands the range of co-doping ions for forming electron-pinned defect-dipole clusters, providing a promising avenue for further development of CP as well as advancing the defect chemistry.

Generally speaking, this work presents more facts that assist to reveal a real trend to guide the design and development of materials of this type. Scientifically, it provides further evidence to confirm the importance of the EPDD for achieving the colossal permittivity and also reveals the differences in the formation of EPDDs in the same host material (TiO_2_) due to bivalent acceptor Mg and pentavalent donor Ta. This paper therefore is passing critical information on the readers with regard to the development of the colossal permittivity materials and other defect-engineered functional materials, inspiring the scientists and engineers to rethink about the defect chemistry in solids. Technically, colossal permittivity materials have many important applications in electronics but their development has generally been obscured due to the difficulty in achieving a relatively low dielectric loss. Similar to the dielectric properties observed in (In,Ta) co-doped rutile TiO_2_^4^, this material system exhibits high performance colossal permittivity with similar low dielectric loss compared with other reported results in co-doped rutile TiO_2_.

Furthermore, it is noteworthy that the colossal permittivity materials design based on the EPDDs have been extended to other host materials systems, such as perovskite materials^10, 17, 18, 48^. It is timely important to draw people attention on the complexity and difference of defect design in solids.

## Experimental section

Mg + Ta co-doped TiO_2_ samples, i.e. (Mg_1/3_Ta_2/3_)_*x*_Ti_1−*x*_O_2_ with nominal composition of *x* = 0.5%, 5% (denoted 0.5% and 5% Mg + Ta co-doped rutile TiO_2_), and Ta-only doped rutile TiO_2_ [Ta_*x*_(Ti^4+^,Ti^3+^)_1−*x*_O_2_] (where 0.33% of Ti was replaced by Ta^5+^, corresponding to x = 0.33%) ceramics were synthesized by a conventional solid state reaction method where the raw materials TiO_2_ (99.99%, Aldrich), Ta_2_O_5_ (99.99%, Stanford Materials) and MgO (99.99%, Aldrich) were mixed and pressed into pellets, following heat treatments at various temperatures to achieve dense and well crystalline ceramic samples. The average structure of the as-synthesized samples was studied using X-ray powder diffraction (XRD) (PANalytical Empyrean). The microstructure, element distribution and chemical composition were determined by a scanning electron microscopy (SEM) (Hitachi 4300SE/N) with an attached energy dispersive spectrometer (EDS). The electron paramagnetic resonance (EPR) and X-ray photoelectron spectroscopy (XPS) techniques were used to determine the valence of the ions and associated defect state. XPS spectra were fitted by using mixed 80Gaussian-20Lorentzian functions with a Shirley background subtraction. For the dielectric property measurements, all ceramic pellets were coated by silver paste and then heat treated at 800 K for 30 min to form electrodes with a good electrical contact. Sputtered Pt electrodes were also used for the comparison. The frequency (20 Hz ≤ *f* ≤ 1 MHz) and temperature (10 K ≤ T ≤ 450 K) dependence of the dielectric behavior of the ceramic pellets were studied using a closed-cycle refrigerator in conjunction with a precision LCR meter (Agilent E4980A). Dielectric frequency spectra in the frequency range 40 Hz to 10 MHz were measured with an LCR meter (Agilent 4294 A) at room temperature. The resistance of the samples was measured using Keithley 6517B Electrometer/High Resistance Meter (Maximum Output Voltage 100 V). All DFT calculations were performed using the PAW formalism within the VASP code. A plane wave energy cutoff of 400 eV and a 2 × 2 × 2 Monkhorst-Pack k-point grid was used. The Ti, O, Mg and Ta potentials treated 12, 6, 10 and 11 electrons as valence, respectively. The PBE + U functional was used, with effective U parameters of 7.4 eV for Ti and Ta d electrons and 5.4 eV for O p electrons. The calculations were spin polarized and oxidation states were assessed by considering the spin density within spheres centered on the ions.

### Data availability statement

Supporting information and all other data are available online.

## Electronic supplementary material

Supporting information
