# Supplementary material for: Colossal permittivity behavior and its origin in rutile (Mg1/3Ta2/3)xTi1-xO2
Source: Sci Rep. 2017 Aug 30;7:9950. doi: 10.1038/s41598-017-08992-x (PMC5577065; doi:10.1038/s41598-017-08992-x)
Supplement: Supplementary file 1 — Supporting information [file 41598_2017_8992_MOESM1_ESM.pdf]

**Supporting information**

# Colossal permittivity behavior and its origin in rutile $(Mg_{1/3}Ta_{2/3})_xTi_{1-x}O_2$

Wen Dong[1], Dehong Chen[1], Wanbiao Hu[1], Terry J. Frankcombe[2,*], Hua Chen[3], Chao Zhou[4], Zhenxiao Fu[4], Xiaoyong Wei[5], Zhuo Xu[5], Zhifu Liu[6], Yongxiang Li[6] and Yun Liu[1,*]

[1]Research School of Chemistry, the Australian National University, ACT 2601, Australia

[2]School of Physical, Environmental and Mathematical Sciences, The University of NewSouth Wales, Canberra, ACT 2601, Australia.

[3]Centre for Advanced Microscopy, The Australian National University, ACT 2601, Australia

[4]Fenghua Advanced Technology Holding Co. Ltd., China.

[5]Electronic Materials Research Laboratory, Key Laboratory of the Ministry of Education & International Centre for Dielectric Research, Xi'an Jiaotong University, Xi'an 710049, China

[6]CAS Key Lab of Inorganic Functional Materials and Devices, Shanghai Institute of Ceramics, Chinese Academy of Sciences, Shanghai 200050, China.

*To whom the correspondence should be addressed: yun.liu@anu.edu.au, t.frankcombe@adfa.edu.au

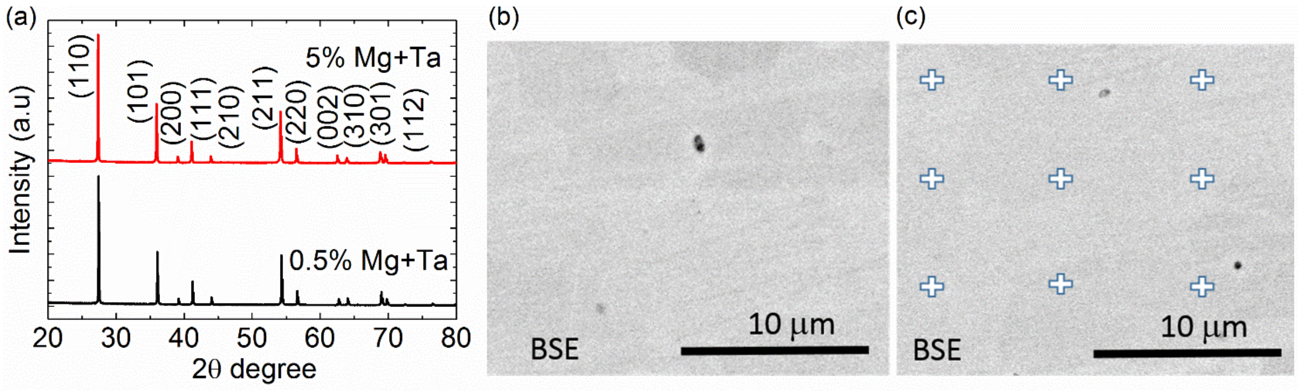


**Figure S1 (a) XRD patterns of 0.5% and 5% Mg+Ta co-doped rutile $TiO_2$. (b) The back scattering electron (BSE) images of Mg+Ta co-doped $TiO_2$ with nominal co-doping levels of 0.5%. (c) Same as (b), with 5% co-doping.** The white "+" symbols labeled in the images are the nine points that are used to collect EDS spectra to determine the average Mg:Ta:Ti ratio to the total cation, and the homogeneity of the element distribution. The BSE images do not show any inhomogeneous element distribution for those samples in the measureable resolution. The EDS spectra were collected at nine points to indicate the differentiation of Mg, Ta and Ti distribution and experimentally determine the average Mg:Ta:Ti ratio to the total cation. Note that since the detected element distribution for the sample 0.5% co-doping level is within the error limit range of the EDS technique, the resultant In:Ta ratios for the samples with co-doping level of 5% are only presented, which are 0.015(±0.001):0.033(±0.001):0.95(±0.001), clearly showing the average ratio is in a good agreement with the nominal ratio of (0.05/3):(0.1/3):0.95. There is no inhomogeneous chemical distribution observed in any of the samples.

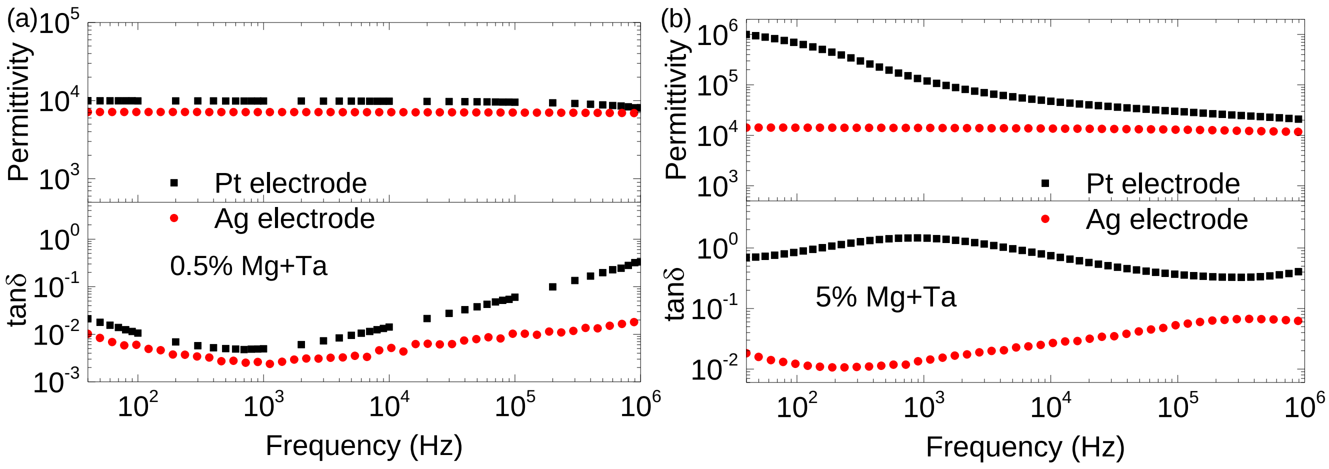


**Figure S2 Frequency dependent dielectric permittivity and loss (tan δ) for 0.5% and 5% Mg+Ta co-doped rutile $TiO_2$ with Pt or Ag as electrode, respectively**. The dielectric permittivity of 5% Mg+Ta co-doped $TiO_2$ varies very significantly, showing a strong dependency on electrodes. The lower 0.5% co-doped $TiO_2$ shows a relatively less electrode-dependency. Indeed, it is found that the dielectric loss increases when the Pt replaces Ag as electrodes. In comparison with significant dielectric loss change in 5% samples, we can conclude that surface layer capacitor's contribution exists but not be predominated in the case of 0.5% Mg+Ta co-doped $TiO_2$.

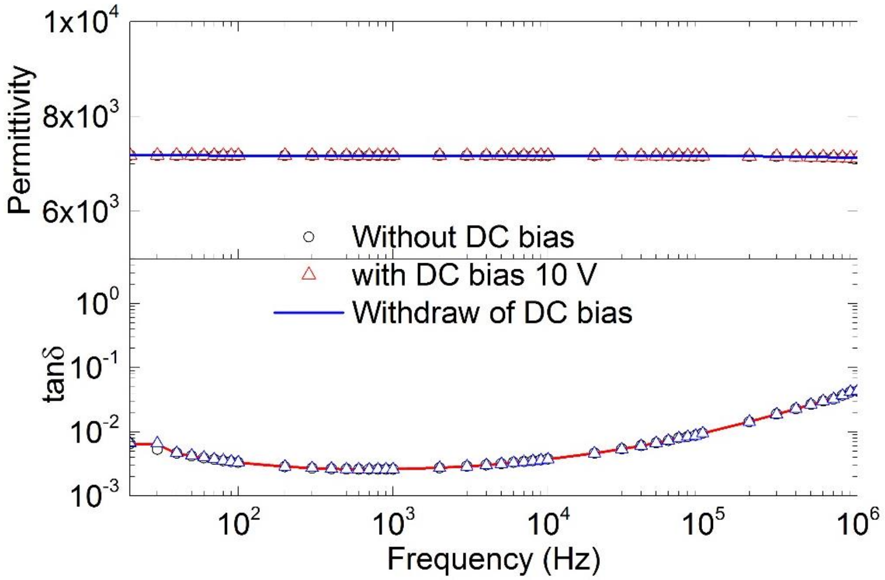


**Figure S3 Frequency dependence of the dielectric properties for 0.5% Mg+Ta co-doped rutile $TiO_2$ with, without, and withdraw of 10 V DC bias voltage.** It is obvious that the measured room temperature, frequency-dependent permittivity and loss tangent curves under 10V DC bias remains nearly unchanged with and without the DC bias, respectively. Thus, there is no noticeable voltage dependency of the permittivity and loss tangent. It differs from grain-boundary, interfacial polarization dominated systems where numerous space charges are accumulated at grain boundaries, resulting a significant increase in both permittivity and loss when a small DC bias is applied[1, 2].

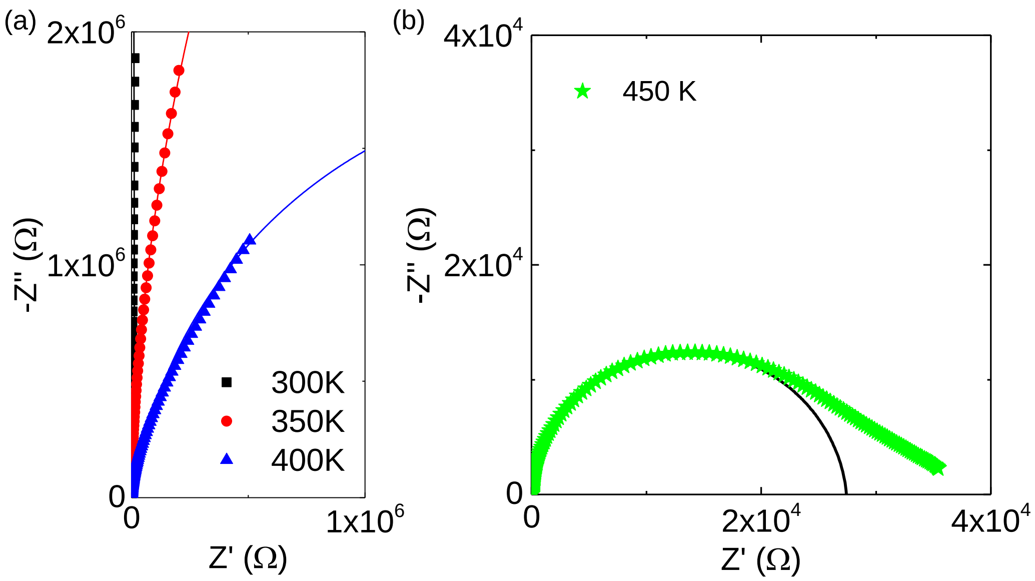


**Figure S4 (a) and (b) complex impedance plots [Z'(ω)-Z"(ω)] (filled symbol) of 0.5% Mg+Ta co-doped rutile $TiO_2$ measured in the frequency range of 20Hz-2MHz from 300 K to 450 K, and corresponding fitting results (solid lines) using Cole-Cole model.** Complex impedance analysis was carried out on 0.5% Mg+Ta co-doped rutile $TiO_2$ to investigate the contribution of interfacial effect to the CP behavior in the system. In the temperature range from 300 K to 400 K, the complex impedance spectrum measured at the three temperature points in Figure S4a can be well fitted using only one parallel RQ element. The fitting results in the temperature range of 300~400 K suggest that the complex impedance contains only one constituent intra grain contribution which can be considered to be the source of the colossal permittivity[3]. While the complex impedance at 450 K obviously cannot be fitted using only one parallel RQ element, but requires two connected in series i.e. $(R_gQ_g)(R_{gb}Q_{gb})$. Here, $R_g$ and $R_{gb}$ represent the grain and grain boundary resistances, respectively. $Q_i$ (g, gb) is the corresponding phase element[4]. The impedance results for 450 K is quite consistent with the temperature-dependent behavior, with the permittivity as well as the dielectric loss rising around this temperature due to interfacial barrier layer capacitor effect. This is in good agreement with the (only high-temperature) Maxwell-Wagner type. Therefore, the CP behavior in the 0.5% Mg+Ta co-doped rutile $TiO_2$ samples should be attributed to the quasi-intrinsic EPDD in grain interior. The significantly lower dielectric loss in this co-doped system indicates that the EPDD effect works more efficiently in localization of electrons from $Ti^{3+}$ than in M+Nb co-doped rutile $TiO_2$ (M=In, Ga, and Zn) [5-7].

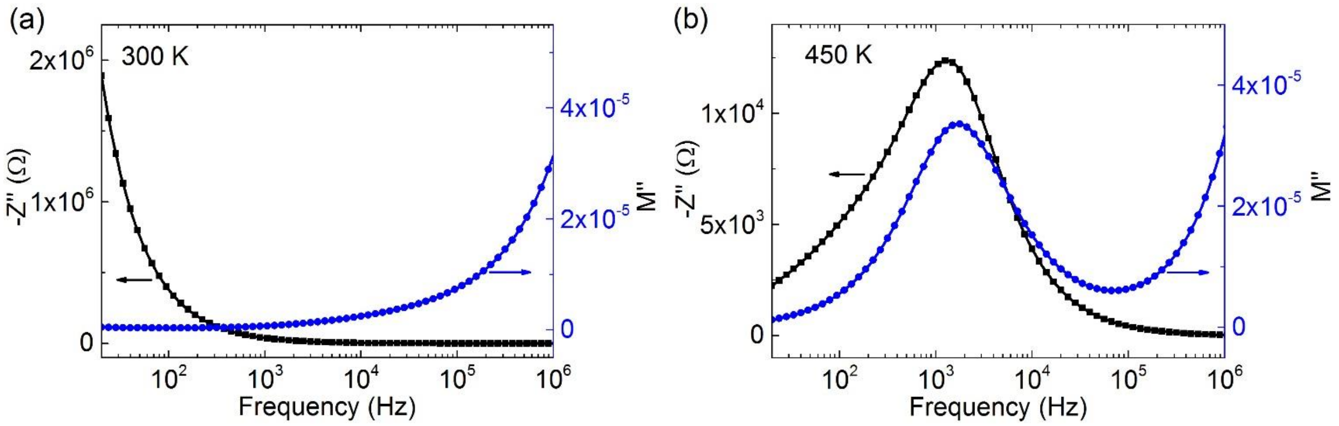


**Figure S5 Imaginary impedance and modulus spectrum as a function of frequency for 0.5% Mg+Ta co-doped rutile $TiO_2$.** In Figure S5, the impedance and modulus analysis suggests that the stable permittivity behaviour over the broad temperature range to be from a highly localized dielectric relaxation, as the relaxation peak on impedance and modulus are rather separated from each other.[8] While at high temperature e.g. at 450 K, localized dielectric relaxation combined with long range conductivity induced interfacial dielectric relaxation evidenced by nearly overlapping peaks of impedance and modulus where electrons are thermally excited and transport to grain boundaries and interfaces. These results suggest that in our case, the high performance CP behaviour to be mainly originated from intragrain contributions rather than interfacial barrier layer capacitor effect.

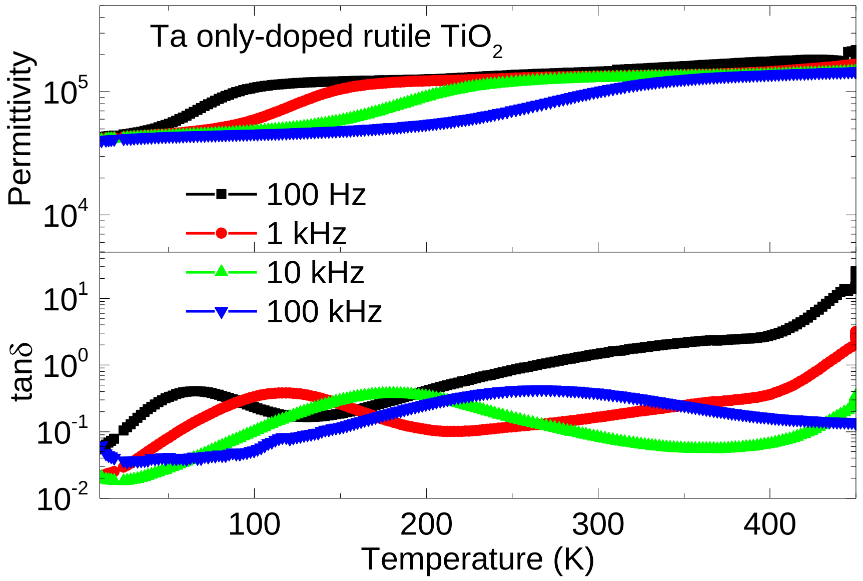


**Figure S6 Temperature dependent dielectric spectra for Ta only doped rutile $TiO_2$, where ~0.33% $Ti^{4+}$ ions were replaced by $Ta^{5+}$.** This spectra show similar relaxations to the co-doped samples in the temperature range from 50 K to over 300 K.

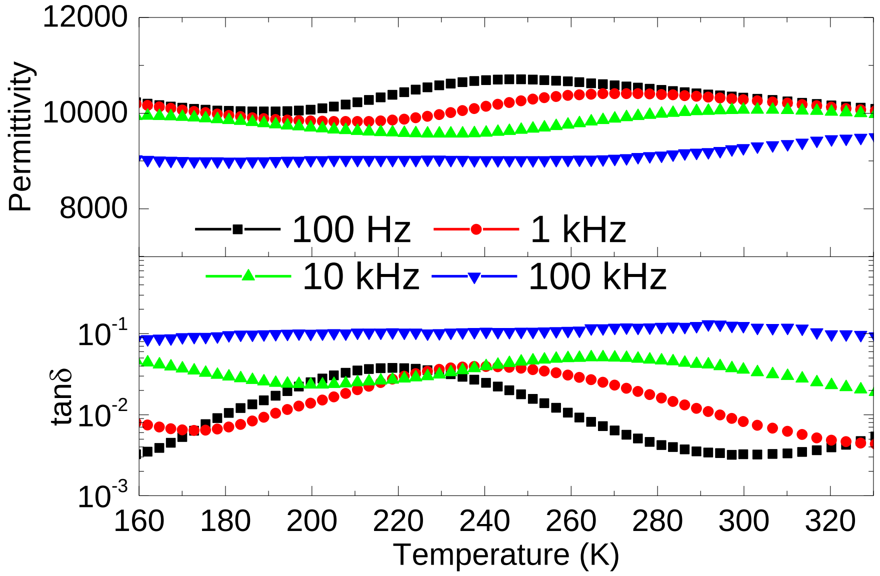


**Figure S7 Temperature dependences of dielectric property for 0.5% Mg+Ta co-doped rutile $TiO_2$ magnified in the temperature region from 160 K to 330 K.** In this graph, significant characteristics of this strong frequency dependent relaxation are the permittivity peaks as well as dielectric loss peaks. The amplitude of the permittivity is within the range of the ionic polarizations.

## References


1. Lunkenheimer, P., Fichtl, R., Ebbinghaus, S. G., Loidl, A., Nonintrinsic origin of the colossal dielectric constants in $CaCu_3Ti_4O_{12}$. *Phys. Rev. B*, **70**, 172102 (2004).
2. Liu, G.-Z., Wang, C., Wang, C.-C., Qiu, J., He, M., Xing, J., Jin, K.-J., Lu, H.-B., Yang, G.-Z., Effects of interfacial polarization on the dielectric properties of $BiFeO_3$ thin film capacitors. *Appl. Phys. Lett.*, **92**, 122903 (2008).
3. Yang, J., He, J., Zhu, J. Y., Bai, W., Sun, L., Meng, X. J., Tang, X. D., Duan, C.-G., Rémiens, D., Qiu, J. H., Chu, J. H., Small polaron migration associated multiple dielectric responses of multiferroic $DyMnO_3$ polycrystal in low temperature region. *Appl. Phys. Lett.*, **101**, 222904 (2012).
4. Haile, S. M., West, D. L., Campbell, J., The role of microstructure and processing on the proton conducting properties of gadolinium-doped barium cerate. *J. Mater. Res.*, **13**, 1576-1595 (1998).
5. Hu, W., Liu, Y., Withers, R. L., Frankcombe, T. J., Norén, L., Snashall, A., Kitchin, M., Smith, P., Gong, B., Chen, H., Schiemer, J., Brink, F., Wong-Leung, J., Electron-pinned defect-dipoles for high-performance colossal permittivity materials. *Nat. Mater.*, **12**, 821-826 (2013).
6. Dong, W., Hu, W., Berlie, A., Lau, K., Chen, H., Withers, R. L., Liu, Y., Colossal dielectric behavior of Ga+Nb co-doped rutile $TiO_2$. *ACS Appl. Mater. Interfaces*, **7**, 25321-25325 (2015).
7. Wei, X., Jie, W., Yang, Z., Zheng, F., Zeng, H., Liu, Y., Hao, J., Colossal permittivity properties of Zn,Nb co-doped $TiO_2$ with different phase structures. *J. Mater. Chem. C*, **3**, 11005-11010 (2015).

8. Gerhardt, R., Impedance and dielectric spectroscopy revisited: Distinguishing localized relaxation from long-range conductivity. *J Phys. Chem. Solids*, **55**, 1491-1506 (1994).
